# Supplementary material for: Marsupial and monotreme milk—a review of its nutrient and immune properties
Source: PeerJ. 2020 Jun 23;8:e9335. doi: 10.7717/peerj.9335 (PMC7319036; doi:10.7717/peerj.9335)
Supplement: Supplemental Information 1 [file peerj-08-9335-s001.docx]

Supplementary Table 1. Macronutrient compositions (g/100mL) of tammar wallaby and eastern quoll milk

| Species | Diet | Time of lactation (weeks postpartum) | Weaning begins (weeks) | Protein | Lipid | Carbohydrate |
| --- | --- | --- | --- | --- | --- | --- |
| Tammar wallaby (*Notamacropus eugenii*) | Herbivore | 10-13 | 39 | 4 | 2 | 8.8 |
|  |  | 20-26 |  | 4.5 | 4 | 11.9 |
|  |  | 30-31 |  | 7 | 11 | 10.1 |
|  |  | 36-38 |  | 13 | 23 | 1.7 |
| Eastern quoll  (*Dasyurus viverrinus*) | Carnivore | 7 | 20 | 5 | 8 | 5.5 |
|  |  | 10 |  | 10 | 17 | 5 |
|  |  | 16 |  | 9 | 11 | 4.5 |
|  |  | 20 |  | 8.5 | 14.5 | 4 |

(1-4)

1. Green B, Griffiths M, Leckie RM. Qualitative and quantitative changes in milk fat during lactation in the tammar wallaby (*Macropus eugenii*). Australian Journal of Biological Sciences. 1983;36(6):455-62.

2. Green B, Merchant J, Newgrain K. Lactational energetics of a marsupial carnivore, the eastern quoll (*Dasyurus viverrinus*). Australian Journal of Zoology. 1997;45(3):295-306.

3. Green B, Newgrain K, Merchant J. Changes in milk composition during lactation in the tammar wallaby (*Macropus eugenii*). Australian Journal of Biological Sciences. 1980;33(1):35-42.

4. Messer M, Green B. Milk carbohydrates of marsupials. II. Quantitative and qualitative changes in milk carbohydrates during lactation in the tammar wallaby (*Macropus eugenii*). Australian Journal of Biological Sciences. 1979;32(6):519-31.
